# Mechanism Underlying Flow Velocity and Its Corresponding Influence on the Growth of *Euglena gracilis*, a Dominant Bloom Species in Reservoirs

**DOI:** 10.3390/ijerph16234641

**Published:** 2019-11-22

**Authors:** Yi Tan, Jia Li, Linglei Zhang, Min Chen, Yaowen Zhang, Ruidong An

**Affiliations:** 1 Institute of Ecology and Environment, State Key Laboratory of Hydraulics and Mountain River Engineering, College of Water Resource & Hydropower, Sichuan University, Chengdu 610065, China; tanyi_scu@163.com (Y.T.); lijia@scu.edu.cn (J.L.); anruidong@scu.edu.cn (R.A.); 2 Department of Architectural Engineering, Urban Vocational College of Sichuan, Chengdu 610110, China; zyw1024@hotmail.com

**Keywords:** hydrodynamic condition, flow velocity, *Euglena gracilis*, algae growth, nutrient absorbance, oxidation level, antioxidant enzyme, optimum flow velocity, stress flow velocity

## Abstract

The effects of hydrodynamics on algae growth have received considerable attention, and flow velocity is one of the most frequently discussed factors. For *Euglena gracilis*, which aggregates resources and is highly resistant to environmental changes, the mechanism underlying the impact of flow velocity on its growth is poorly understood. Experiments were conducted to examine the response of algae growth to different velocities, and several enzymes were tested to determine their physiological mechanisms. Significant differences in the growth of *E. gracilis* were found at different flow velocities, and this phenomenon is unique compared to the growth of other algal species. With increasing flow velocity and time, the growth of *E. gracilis* is gradually inhibited. In particular, we found that the pioneer enzyme is peroxidase (POD) and that the main antioxidant enzyme is catalase (CAT) when *E. gracilis* experiences flow velocity stress. Hysteresis between total phosphorus (TP) consumption and alkaline phosphatase (AKP) synthesis was observed. Under experimental control conditions, the results indicate that flow velocities above 0.1 m/s may inhibit growth and that *E. gracilis* prefers a relatively slow or even static flow velocity, and this finding could be beneficial for the control of *E. gracilis* blooms.

## 1. Introduction

Algal blooms are most likely to occur in lakes and reservoirs because of their low flow velocity, long hydraulic retention time, and low self-cleaning capability [1,2]. Many scholars have explored the relationship between algae growth and factors such as water temperature [3], underground light [4], nutrients [5] and hydrodynamic conditions [6,7,8,9,10,11,12,13]. Among these external factors, hydrodynamic conditions are preferred by scholars because of their ease of regulation and control, especially in reservoirs. The effects of the flow velocity on algae growth, as the most intuitive representation of water flow and the most frequently discussed element, are generally manifested by low flow velocities that promote growth and high flow velocities that inhibit growth [14,15].

Many previous studies have focused on the mechanism underlying the influence of flow velocity on cyanobacteria, green algae, and diatoms. Algae have a range of adaptations to flow velocity, and the adaptation ranges and fitness levels of different algae species are not consistent. At a certain flow velocity, the most suitable algae species can effectively grow and become the dominant species, and the flow velocity is associated with the optimum and limiting conditions for algal growth, namely, the dominant flow velocity and the stress flow velocity, respectively. For example, Song et al. [16] showed that the optimal flow velocity for *Microcystis aeruginosa* was 0.24 m/s. Mcintire et al. [17] found that diatoms were generally the dominant species at high flow velocities, and in slow-moving streams, *Stigeoclonium*, *Oedocladium,* and *Tribonema* often became dominant species. However, the mechanisms underlying flow velocity conditions for *Euglena gracilis* have rarely been mentioned. Scholars [18,19,20,21] have found that *Euglena* has a strong ability to aggregate resources and is highly resistant to extremely harsh environmental conditions. Additionally, *Euglena* was found to have strong swimming ability due to its flagella [22,23,24,25,26,27]. Based on these characteristics, the mechanisms underlying the influence of flow velocity on *E. gracilis* would be quite different from those of other algae species studied in the past.

Analysing year-long samples, Zhang et al. [28] found *E. gracilis* to be one of the representative dominant algae species in the Zipingpu Reservoir, Chengdu. In recent years, there have been reports of *Euglena* blooms occurring in other places. A string of reports on *Euglena* water blooms in various parts of the world have begun to appear, such as in 17 states in the United States [29,30], Poland (in a shallow oxbow lake) [31], Iran (Gorgan Bay) [32], Brazil (Guaiba Lake and the Bacanga River Estuary) [33,34], and China (Luoma Lake and shallow urban lakes in Wuhan) [35,36]. In retrospect, previous research has generally discussed the effects of other algae on nutrient salt consumption and physiological mechanisms. For example, Borchardt [37] found that the growth of *Spirogyra fluviatilis Hilse* was promoted by the diffusion of nutrients and the absorption of nitrogen and phosphorus in algal cell membranes at a flow velocity of 0.12 m/s. Song et al. [16] showed that *M. aeruginosa* has the highest nutrient consumption rate when the flow velocity is 0.3 m/s. In addition, the main functional enzymes involved in the antioxidant reactions among different algae are also different. For example, Song et al. [16] showed that catalase (CAT) is the main functional enzyme of the antioxidant system response of *M. aeruginosa*. Fang et al. [38] found that peroxidase (POD) is the major functional enzyme of green algae in the antioxidant system in response to dioxin-like pollutant toxicity. Obviously, the results indicated that the effects and response mechanisms of different algae species are not the same. Therefore, the mechanisms underlying the influence of the flow velocity on other algae were not applicable to *Euglena*. To better understand the effect of flow velocity on the growth characteristics of *Euglena*, we also evaluated alkaline phosphatase (AKP) based on the phosphorus-AKP metabolic process [39,40,41]. According to instructive conclusions from previous studies, the details on the mechanisms of the impacts from the underlying flow velocity conditions on *E. gracilis* are uncertain and insufficient. In addition, *E. gracilis* blooms have the potential to cause imbalances in the ecosystem and damage to the aquatic environment. Therefore, it is necessary to ascertain how the flow velocity affects the growth of *Euglena* to prevent the occurrence of *Euglena* blooms.

The purpose of this research is to address the mechanisms underlying the effect of flow velocity on *E. gracilis* growth and the corresponding influence of *E. gracilis* on flow velocity stress and strategic support of the antioxidant system and phosphorus-AKP metabolic process. Therefore, this study explores the growth and corresponding growth mechanisms of *E. gracilis* through the exploration of its internal physiology and external environmental indicators. Additionally, the specific effects of different flow velocity conditions on *E. gracilis* are discussed. Therefore, understanding the influencing mechanisms and establishing a flow velocity threshold system can not only provide a reference for predictive warning and risk control of water blooms but also lay a theoretical foundation for the control of algal blooms via reservoir operations.

## 2. Materials and Methods

### 2.1. Algae and Experimental Apparatus

**Figure 1:**
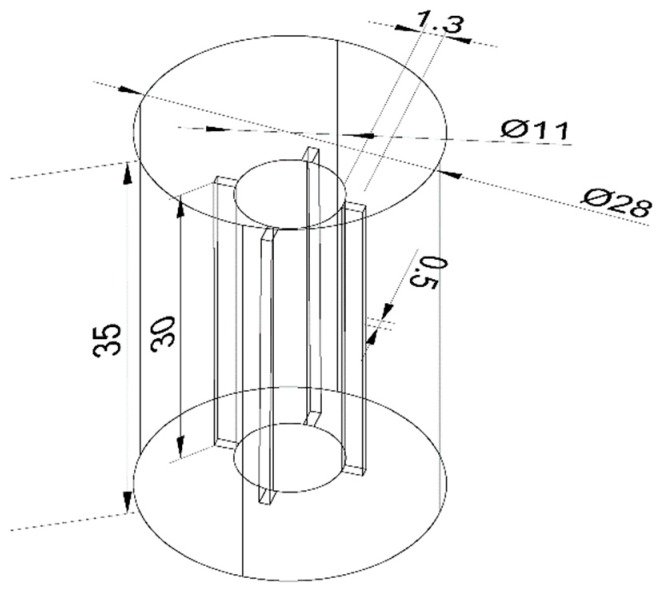
View of the experimental apparatus.

*Euglena gracilis* FACHB-848 was obtained from the Freshwater Algae Culture Collection at the Institute of Hydrobiology (Wuhan, China). Healthy algae were equally (0.8–1 × 10^8^ cells/L) transferred into 6 apparatuses that were filled with 10 L of HUT culture medium. Each device was constructed from polymethyl methacrylate and included a concentric cylindrical sleeve. The diameter of the outer cylinder was 28 cm, and the height was 35 cm. The diameter of the inner cylinder was 11 cm, and it had 4 vanes (1.3 × 0.5 cm) that were 2.7 cm from the bottom. The inner cylinder was filled with moderate pumice stones to prevent floating. The vanes were rotated with a motor that was connected to the inner cylinder to create hydrodynamic conditions and achieve different flow velocities in each device. A typical device is shown in Figure 1.

### 2.2. Experimental Design and Analytical Methods

The velocities were 0, 0.1, 0.2, 0.3, 0.4, and 0.5 m/s for the 6 devices. Algae at a flow velocity of 0 m/s was regarded as the control group. The temperature of the medium was controlled at 25 °C. The selected photoperiod was 12 h of light and 12 h of dark. In addition, the light intensity was set at 2000 Lx for the 24-day experiment.

A sample of 50 mL of algae solution was extracted from each experimental group every 4 days for further testing. Three independent experiments were performed for each treatment. The algal biomass was determined following the method of Chaudhuri et al. [42]. The growth parameter was chlorophyll a (Chla), and external water quality parameters included total nitrogen (TN) and total phosphorus (TP). Chl-a was extracted by using acetone and then measured on a spectrophotometer (TU-1950, PERSEE, Beijing, China) [16,43,44]. After separating the algal cells in the algae solution, the digested samples were used to determine the concentrations of TN and TP. TN was determined by the alkaline potassium persulfate digestion-UV spectrophotometric method (TU-1950, PERSEE, Beijing, China), and TP was determined by ammonium molybdate spectrophotometry (TU-1950, PERSEE, Beijing, China) [16,43,44,45,46]. The above indicators were measured following the method of China’s national standard [44]. A portable pH meter (PHS-2F, Shanghai Yitian Precision Instrument Co. LTD, Shanghai, China) was used to measure pH [16]. To explore the algal growth characteristics, we defined several parameters.

(1) The algal growth rate was determined following the method of Yang Song et al. [16] and is defined as

(1) $$\mu =\frac{\ln X_{t}-\ln X_{t0}}{t}$$

where X_t_ is the maximum biomass of the logarithmic growth phase (cells/L), X_t0_ is the initial biomass at the beginning of the logarithmic growth phase (cells/L), and t is the number of days of the logarithmic growth phase (d).

(2) Nutrient consumption rate is defined as

(2) $$\beta =\frac{X_{24}-X_{0}}{24}$$

where X_24_ is the nutrient concentration at the end of the experiment, X_0_ is the initial nutrient concentration (mg/L), and 24 is the period.

(3) The instantaneous nutrient consumption rate is defined as

(3) $$\alpha =\frac{X_{4}-X_{0}}{4}$$

where X_4_ is the nutrient concentration on the 4th day of the experiment, X_0_ is the initial nutrient concentration (mg/L), and 4 is the first four days.

Lipid peroxidation was measured by the estimation of the malondialdehyde (MDA) concentration, which is the major reactive material of thiobarbituric acid (TBA). The superoxide dismutase (SOD) activity was assessed via the inhibition of nitroblue tetrazolium (NBT). The CAT activity was determined by the decomposition of hydrogen peroxide. The peroxidase (POD) activity was assessed by the formation of tetraguaiacol. AKP synthase activity was determined by the formation of quinonoids. The above indicators were measured using commercial kits purchased from Nanjing Jianchen Bioengineering Institute in China. The commercial kit names are the Malondialdehyde (MDA) assay kit, the Total Superoxide Dismutase (T-SOD) assay kit, the Peroxidase assay kit, the Catalase (CAT) assay kit, and the Alkaline phosphatase assay kit.

### 2.3. Statistical Analysis

The data were reported as the means ± SDs, and the standard deviations for the different batches were <30%. Pearson correlation analysis of the measured parameters was conducted. The assumptions of normal distribution and homogeneity of variance were tested with the Kolmogorov–Smirnov test and Levene’s test, respectively. One-way analysis of variance (ANOVA) and Duncan’s multiple range test (DMRT) were performed to test for the significant differences between treatments for each group under each particular time. For all statistical analyses, a *p*-value of 0.05 was considered significant. All statistical analyses were performed using SPSS 23.0 for Windows (IBM Inc., Chicago, IL, USA).

## 3. Results and Discussion

### 3.1. Changes in the Growth Status of Euglena at Different Flow Velocities

Biomass and Chla were measured simultaneously, and each group was significantly correlated (Table 1, Figure S1, Supplemental Material). Additionally, the determination of the biomass and Chla in laboratory waters was equally effective in reflecting the amount of algae and the water quality. To avoid the redundancy of subsequent analyses, the following analysis and discussion focus on the biomass.

**Figure 2:**
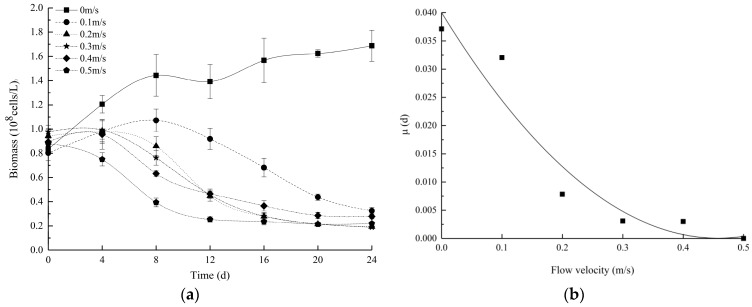
(**a**) Changes in the algal biomass under different flow velocities. (**b**) The specific growth rates of the algae biomass under different flow velocities. All values are the means of triplicate measurements ± SDs (n = 3).

As shown in Figure 2a, the algae biomass of the control group increased the most, with a maximum algal biomass of 1.72 × 10^8^ cells/L. The biomass of the 0.5 m/s group decreased with time, and those of the other experimental groups initially increased and then decreased with time. The growth of the experimental groups was significantly less than that of the control group (Table S1, Supplemental Material). On the fourth day, the control group and the 0.1 m/s group entered the logarithmic growth stage. The biomass of the other experimental groups began to decrease, which indicated that growth was inhibited and that the conditions were not conducive to *E. gracilis* growth under hydrodynamic conditions. Moreover, the higher the flow velocity was, the lower the growth rate of the algae became (Figure 2b). The growth rate of the control group was the largest, and the lowest growth rate was observed for the 0.5 m/s group, in which flow velocities above 0.1 m/s gradually inhibited the growth of *E. gracilis*.

At the end of the experiment, the biomass of the control group was significantly higher than the biomasses of the experimental groups (*p* < 0.05) throughout the experiment (Table S1, Supplemental Material). Based on regression analysis, an empirical formula (R^2^ = 0.9263) of the relationship between the biomass growth rate and flow velocity was obtained according to Figure 2b.

(4) $$\mu =0.1907v^{2}-0.1746v+0.04$$

where *μ* is the biomass growth rate and *v* is the flow velocity. Based on regression analysis, the unfavourable flow velocity for *E. gracilis* was 0.46 m/s, and the 0 m/s hydrostatic condition increased algal growth and caused an obvious increase in the biomass.

This result is different from the previous conclusion [15,16] that appropriate velocities promote algae growth and that there is a positive effect limit. Over the 24-day observation period, we concluded that *E. gracilis* prefers 0 m/s.

The polynomial fitting of the entire cycle of the algae biomass curve was performed to obtain the empirical formula for the prediction of *E. gracilis* growth at different flow velocities (Table 2). The correlation coefficient of each formula remained high, indicating that the formulas effectively mathematically expressed the growth process of *E. gracilis* under different flow velocities during the experimental period. This set of formulas can provide a reference for the initial prediction of the algae bloom trend in reservoirs. Due to the limitations of experimental equipment, operating conditions, cycles, etc., the applicability of these formulas must be further verified.

According to the calculations, the shear stress of the 0.5 m/s group reached 0.0104 Pa, and a previous study [16] showed that shear stresses above 0.0043 Pa may damage algae, which may indicate that the same effect as observed by Song et al. [16] was identified in this study. However, more obvious differences caused by the different flow velocities were detected between Days 4–8 and Days 8–20 throughout the experiment. Thus, it can be inferred that the flow velocity effect on *E. gracilis* growth is time cumulative, and the appropriate flow velocity could shorten the algal lifecycle relative to static water.

### 3.2. Changes in Lipid Oxidation and the Antioxidant Enzyme Activity of Euglena at Different Flow Velocities

**Figure 3:**
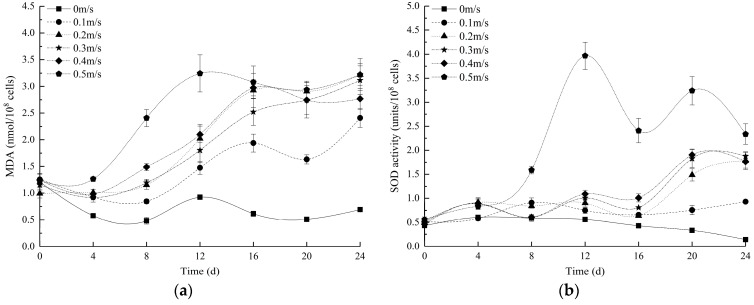

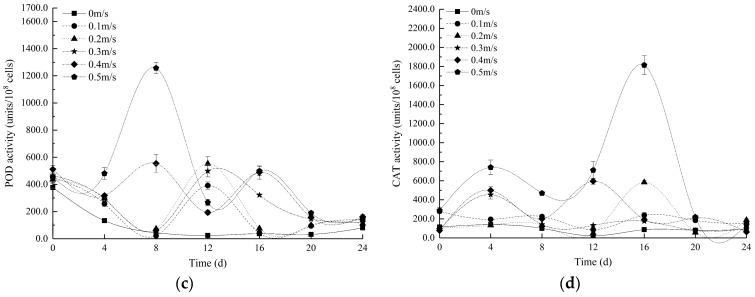
Changes in (**a**) malondialdehyde (MDA), (**b**) superoxide dismutase (SOD), (**c**) catalase (CAT), and (**d**) peroxidase (POD) at different flow velocities. All values are the means of triplicate measurements ± SDs (n = 3).

Figure 3 shows the changes in the MDA content and SOD, POD, and CAT activities at different flow velocities over time. Each group exhibited an obvious peak after the 8th day. Overall, the MDA content of the control group was the lowest, followed by that of the 0.1 m/s group. The SOD activity of each experimental group increased to different degrees throughout the experiment, and that of the 0.5 m/s group began to rise sharply on the 8th day. After the peak appeared on the 12th day, the activity level decreased. Similarly, the SOD activity of the control group was the lowest, followed by that of the 0.1 m/s group.

The POD activity of each group (except the 0.4 and 0.5 m/s groups) decreased in the first 8 days and then increased and peaked on the 12th day before returning to a normal level on the 20th day. The POD activity of the 0.4 and 0.5 m/s groups decreased first and then increased in the first 8 days, peaked on the 8th day, displayed a slightly smaller peak on the 16th day, and returned to normal levels on the 20th day. The CAT levels of the control group and the 0.1 m/s group changed smoothly throughout the experimental period, and the CAT levels of the other experimental groups increased to different degrees in the first 16 days and then stabilized on the 20th day and remained stable until the end of the experiment. The CAT level of the 0.5 m/s group continued to rise. Specifically, a maximum value appeared on the fourth day, followed by a peak on the 16th day and an eventual steep drop before stabilizing.

In general, the production and elimination of reactive oxygen species (ROS) in the cells reflect a state of dynamic equilibrium, and stress conditions can break this balance [47], causing damage to the cell structure and functions [48]. As a major product of lipid peroxidation and an effective indicator of cellular oxidation levels, MDA indirectly reflects the cellular oxidation level [49]. Similarly, the three major enzymes (SOD, POD, and CAT) that comprise the antioxidant defence system and scavenge ROS are also effective indicators of cellular oxidation levels [47,48]. The MDA contents of the experimental groups (except for the 0 m/s group) remained stable at the beginning of the experiment. On the fourth day, the flow stress caused the accumulation of ROS in the cells, which resulted in simultaneous antioxidant system activity and stimulated the algae to produce a large amount of antioxidant enzymes to resist the damage caused by ROS accumulation in the body. Then, over time, the ROS in the *Euglena* cells accumulated in large amounts, and the MDA content also increased gradually. On the 12th day, the MDA content peaked, as did the activities of SOD, POD, and CAT. All the indexes of the experimental group were significantly higher than those of the control group (*p* < 0.05), indicating that the algae in the experiment preferred relatively static water for optimal growth and that the flow velocity level was the most stressful factor that influenced the algae within the experimental flow velocity range (Tables S2–S5, Supplemental Material).

Table 3 shows that the correlation between the MDA content and biomass of each group increases with the flow velocity, with a significant negative correlation, and that the correlation coefficient varies from small to large (Figures S2 and S3, Supplemental Material). Notably, the production of free radicals in algae cells is closely related to exogenous stress [50], which may be due to flow velocity-based stress conditions. Additionally, the correlation between the MDA content and SOD activity of each group increased with the flow velocity, and the correlation coefficient varied from small to large. This finding indicates that the more pronounced the flow stress is, the more rapid the responses of SOD to MDA in algal cells.

Furthermore, we found that the synergistic and co-operative interactions among antioxidants depended on the sequential degradation of peroxides and free radicals and the mutual protection of enzymes, which was supported by the conclusions of Kumar et al. [51]. This synergy is enhanced by the mutual protection of antioxidant enzymes. Taking the 0.5 m/s group as an example, under flow velocity stress, the antioxidant system in the *Euglena* cells first responded to MDA with CAT (Day 4) and POD (Day 8). Then, the SOD levels increased, and the activity peaked on the 12th day. CAT also responded at the beginning of the experiment (Day 4), but the CAT activity decreased on the 8th day, potentially because of superoxide inactivation due to insufficient SOD availability. This theory is consistent with the conclusion of Blum and Fridovich [52], who found that SOD protects CAT from the inactivation of superoxide. After the SOD level peaked on the 12th day, the CAT level peaked on the 16th day, which was supported by the above point. On the 16th day, CAT protected SOD from the inactivation of hydroperoxides, which was consistent with the conclusions obtained by Sinet and Garber [53]. Subsequently, the CAT level decreased (Days 16–20) until it stabilized at Days 20–24, and SOD slowly increased (Days 16–20) before declining on Days 20–24, confirming the above view. In addition, significance analysis indicated that there were significant differences (*p* < 0.05) in the changes in the four physiological indicators between the control and experimental groups (Tables S2–S5, Supplemental Material). The MDA content and antioxidant enzyme activity in the 0.5 m/s group were higher than those in the other groups after the 8th day, indicating that the production and accumulation of ROS at this flow velocity were the highest and that the algae cells increased the antioxidant enzyme activity to resist oxidative damage and maintain normal growth. At the end of the experiment, the high levels of all antioxidant enzymes in the 0.5 m/s group indicated that the flow velocity still significantly stimulated the activity of the antioxidant mechanism. The biomass of the 0.5 m/s group was still at a considerable amount, which may indicate that the resistance mechanism in the algae cells of *E. gracilis* was still working at 0.5 m/s. From the first day to the 24th day, the MDA content of the 0 m/s group was always lower than that of the other groups, and the ROS level was low, which may be an intrinsic physiological reason for the growth of algae cells under the dominant flow velocity. Compared to the results of Song et al. [16], we found that *E. gracilis* exhibits a relatively lower MDA content than that in *Microcystis aeruginosa* under the same flow velocity conditions, potentially because *E. gracilis* is highly resistant to environmental changes. In contrast, antioxidant enzyme activity responds to flow velocity stress, and CAT is the main antioxidant enzyme used for defence [16]. Alternatively, POD, as a pioneer enzyme, is the first to defend (e.g., SOD displayed a pioneer enzyme in *M. aeruginosa* [16]). This enzyme mechanism for coping with flow stress is very different, which may be the internal reason why *E. gracilis* has a strong endurance to flow velocity stress conditions.

### 3.3. Changes in the Nutrient Levels and Extracellular Hydrolase Activities in Water at Different Flow Velocities

**Figure 4:**
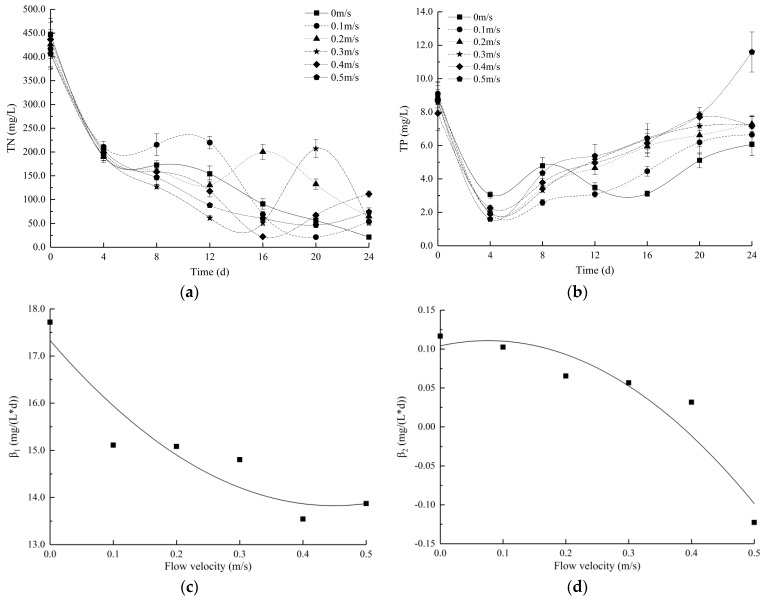
Changes in the (**a**) TN and (**b**) TP concentrations at different flow velocities. The specific consumption rates of (**c**) TN and (**d**) TP concentrations at different flow velocities. All values are the means of triplicate measurements ± SDs (n = 3).

Figure 4a,b show the changes in nutrient concentrations in the medium. Along with algal growth, the nitrogen in each group exhibited a downward trend, and the phosphorus level decreased first and then increased. After the fourth day, TN exhibited a variable level over the N metabolic cycle, indicating that the experimental groups of algae cells were slow to propagate but highly metabolized. The TP level increased to varying degrees. However, the TP content at 0.5 m/s on the 24th was higher than the initial content.

At the end of the experiment, the TN consumption of the control group was significantly higher than that of the experimental groups (*p* < 0.05) throughout the experiment. There was no statistically significant difference in TP consumption in any group except the 0.5 m/s group (*p* > 0.05) (Tables S6 and S7, Supplemental Material). The control group consumed the most phosphorous, followed by the 0.1 m/s group, and the absorption of the other experimental groups was low.

Based on regression analysis, empirical formulas (R_1_^2^ = 0.8773, R_2_^2^ = 0.9077) for the relationship between the nutrient uptake rates and flow velocity were obtained according to Figure 3c,d.

(5) $$\beta_{1}=17.394v^{2}-15.616v+17.32$$

(6) $$\beta_{2}=-1.1652v^{2}+0.1776v+0.1041$$

where β is the nutrient uptake rate and v is the flow velocity. Based on regression analysis, the most unfavourable flow velocity for the absorption of TN nutrients by *E. gracilis* was 0.45 m/s, and the dominant flow velocity for TP nutrient absorption was 0.07 m/s.

The decreases in the TN and TP contents in water were due to the propagation and metabolism of *E. gracilis*. Nitrogen fixation maintains growth metabolism, and phosphorus synthesis sustains physiological tissues and promotes growth, proliferation, and resistance to exogenous stresses [16]. Therefore, it can be inferred that the consumption of TN and TP in the medium effectively reflects the demands of algal growth. Comparing the consumption of TN and TP, the demand for TN was much higher than that for TP.

Table 4 shows the instantaneous nutrient consumption rates and steady-state consumption rates of TN and TP at different flow velocities. The instantaneous nutrient consumption rates are the slopes of nutrient consumption on the fourth day.

Istvànovics et al. [54] found that algae may need to supplement intracellular phosphorus reserves to maintain growth or alleviate a phosphorus deficiency. On the fourth day, the instantaneous consumption rate of TP was higher than the steady-state consumption rate, which indicates that *E. gracilis* consumed more nutrients than it required. *E. gracilis* needs to store a large amount of nutrients before proliferating. In addition, Schoffelen et al. [39] found that freshly assimilated *p* is potentially stored as polyphosphates (PolyPs) and proposed that algae absorb *p* to induce AKP and increase biological competitiveness. PolyP formation and storage has traditionally been viewed as a “luxury”, where uptake occurs under phosphate-replete conditions for use at times of *p* starvation. Thus, at the beginning of the experiment, the high resource aggregation ability of the species was reflected by the absorption of inorganic phosphate (Pi). At the end of the experiment, the instantaneous consumption rates of TP were lower than their steady-state consumption rates. This result may be because the algae simultaneously utilized the nutrients in the water body and self-synthesized PolyP and paramylon. This finding may be consistent with those of Ciugulea and Triemer [55], who noted that *E. gracilis* synthesizes a large number of paramylons for energy storage to maintain growth and survival. The differences among the groups may be because the biomass and growth rate of the 0 m/s group were higher than those of the experimental groups; naturally, the demand for phosphate in the 0 m/s group was correspondingly high.

The TP content of the 0.5 m/s group on the 24th day was higher than the initial content, potentially because the high flow velocity destroyed the cell morphology and disrupted the metabolism of ROS, releasing phosphate compounds that were not fully utilized or self-synthesized in the cell. This result is similar to the conclusions of Song et al., Gao et al., and Wang et al. [16,56,57], who showed that broken algae were most likely to release reactive phosphorus and TP. Gao et al. [56] also found that this nutrient-releasing process could accelerate the decline and decomposition of algae. Figure 4c,d shows that the nutrient absorption rate of the 0 m/s group is the largest. The experimental results indicate that *E. gracilis* behaves differently than *M. aeruginosa* because *M. aeruginosa* has the highest nutrient consumption rate when the flow velocity is 0.3 m/s [16]. The reason for the difference may be because *E. gracilis* prefers a relatively slow or even static flow velocity and highly aggregates resources [21].

**Figure 5:**
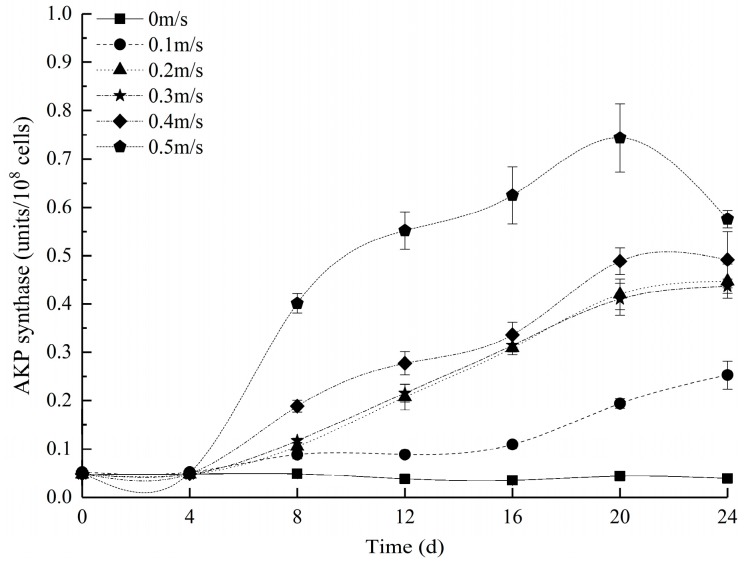
Changes in alkaline phosphatase (AKP) enzymes at different flow velocities. All values are the means of triplicate measurements ± SDs (n = 3).

Figure 5 shows the evolution of AKP enzyme activity over time at different flow velocities. The AKP of the experimental groups remained stable for 4 days and then began to change to various degrees beginning on the 4th day. The larger the flow velocity was, the higher the activity of AKP. The control group remained stable throughout the experimental period, and the AKP content of the group was the lowest (Table S8, Supplemental Material).

AKP is a highly specific phosphate ester hydrolase that catalyses all types of phosphate ester in the hydrolysis process and transfers phosphate groups. AKP is directly involved in phosphorus metabolism, providing a source of phosphorus for rapid growth. In aquatic ecosystems, AKP plays an important role in the conversion of phosphorus [40,41]. Tian et al. [58] found that AKP is involved in catalysis in the hydrolysis of phosphate monoester or transphosphatase in alkaline environments. Moreover, Hoppe [59] reported that the optimum pH for AKP ranged from 8.3 to 9.6.

At the beginning of the experiment (0–4 days), we observed hysteresis between the synthesis of AKP and the utilization and absorption of TP. This finding potentially occurred because the pH of the water body was approximately 7.5 (Table S9, Supplemental Material), the supply of TP was sufficient during this period, and the algae cells were not severely oxidized and damaged. These results may indicate that the water body did not constitute an environment suitable for catalysis with the AKP enzyme. Along with algal growth (4–20 days), we observed that the pH exceeded 8.27 (Table S9, Supplemental Material), the TP slowly increased, and the AKP enzyme gradually increased with increasing flow velocity, while the algal biomass (except for that of the 0 m/s group) exhibited different degrees of decline. Additionally, the algae cells resisted flow velocity stress, and cell metabolism increased, which also stimulated the synthesis of the AKP enzyme. This finding was supported by Schaub [60], who noted that an increase in algae metabolism stimulated the secretion of AKP. Similarly, the significant reduction in TP previously stimulated the secretion of AKP enzymes. Afterwards, the AKP enzyme was involved in catalysis in the hydrolysis of various phosphate monoesters and transphosphorylation, resulting in elevated phosphate levels and increases in the TP content to various degrees. At the end of the experiment (20–24 days), due to the severe damage to the algae cells in the 0.5 m/s group, the synthesis of the AKP enzyme was hindered, resulting in decreased activity [57]. At this time, the algae cells in the 0.5 m/s group were broken, and the release caused the TP content to be higher than the initial value.

All of the above observations indicate that the metabolism and decay of algae can cause the synthesis and secretion of AKP. This effect was supported by Wang et al. [57], who found that the damage to cells may hinder AKP synthesis. In addition, we found that the pH required for catalysis by the AKP enzyme of *E. gracilis* was 8.27, which is not the same as that observed by Li, P. et al. [61], who found that the optimum pH for catalysis by the AKP enzyme of the cyanobacterium *Nostoc flagelliforme* was 11. This relatively low activation mechanism of AKP enzymes also explains the internal physiological reason why *E. gracilis* is more resistant to extreme environmental conditions and how *E. gracilis* aggregates resources under nutrient-limiting conditions.

Table 5 shows that the AKP and algae biomass are significantly negatively correlated with increasing flow velocity and that the correlation coefficient increases with increasing flow velocity. Additionally, AKP is positively correlated with the MDA content and SOD activity, and the correlation coefficient increased with increasing flow velocity.

The formation of algal blooms mainly depends on the short-term surge of algae biomass, and the prevention and control of algae in reservoirs can be carried out based on the internal physiological characteristics of algae. Therefore, determining the inhibition flow velocity threshold for *Euglena* will help to optimize the control of *Euglena* blooms.

Other issues require further study. Under experimental control conditions, the optimum flow velocity of *E. gracilis* was in the range of 0 to 0.1 m/s based on the study results, and more detailed research should be conducted within this range. In addition, limitations of the laboratory contributed to some uncertainty in this study. The experiment simulated an actual running velocity environment of the reservoir, and the flow velocity was the only controlled variable, whereas real-world situations are more complicated, with hydrodynamic conditions such as shearing, turbulence, and blending. All of these issues deserve further study.

## 4. Conclusions

Long hydraulic retention time, low self-cleaning capability, and nutrient enrichment are caused by the low-velocity operations in reservoirs, which create advantageous conditions for the explosion of *E. gracilis* blooms. This study focused on the corresponding mechanisms of the growth characteristics and effects of the flow velocity conditions on *E. gracilis* based on the algal biomass and other specific factors (e.g., oxidation levels, antioxidant enzyme systems, nutrient uptake, and AKP). The results indicated that the effects of flow velocity on *E. gracilis* accumulated with increases in both magnitude and duration. *E. gracilis* prefers a relatively slow or even static flow velocity, and when the flow velocity reached 0.5 m/s, it had a significant inhibitory effect on *E. gracilis*. In addition, the synergistic effect of antioxidant enzymes and the *p* reserves in the early stage and the *p* metabolism mechanism involved in AKP enzymes effectively supported the internal physiological characteristics of *E. gracilis* with high tolerance to resistance to flow velocity stress. These findings provide a basic summary of the effects of the flow velocity on *E. gracilis* growth. In the design of an algae bloom control scheme, the inhibition flow velocity, the internal physiological characteristics of *Euglena*, and the dam discharge should be considered comprehensively to ensure that the flow velocity reaches the inhibition flow velocity threshold. This design can not only ensure the interests of the reservoir but also effectively inhibit the occurrence of *E. gracilis* blooms by regulating the flow velocity intermittently in the reservoir. These findings serve as a beneficial reference for the prediction and supervision of *E. gracilis* blooms in reservoirs.

## Figures and Tables

**Table 1:** Correlations between the algal biomass and Chla under different flow velocities for 24 days.

Flow Velocity (m/s)	Chla
0	0.904 *
0.1	0.822 *
0.2	0.894 *
0.3	0.931 **
0.4	0.878 *
0.5	0.902 *

A *p*-value of 0.05 is considered significant; ** indicates *p* < 0.01, and * indicates *p* < 0.05.

**Table 2:** Prediction formulas for algae growth under different flow velocities for 24 days.

Flow Velocity (m/s)	Prediction Formula of Algal Growth	R^2^
0	y = −0.0015x^2^ + 0.067x + 0.8971	0.9418
0.1	y = −0.0025x^2^ + 0.0328x + 0.8614	0.9172
0.2	y = 0.0004x^2^ – 0.0475x + 1.0554	0.8984
0.3	y = 0.0007x^2^ – 0.055x + 1.0773	0.938
0.4	y = 0.0008x^2^ – 0.0489x + 0.984	0.9238
0.5	y = 0.0019x^2^ – 0.075x + 0.9193	0.9636

**Table 3:** Correlations between MDA and biomass and SOD at different flow velocities for 24 days.

Flow Velocity (m/s)	Biomass	SOD
0	−0.720	−0.020
0.1	−0.877 **	0.333
0.2	−0.985 **	0.645
0.3	−0.962 **	0.817 *
0.4	−0.955 **	0.737
0.5	−0.978 **	0.904 **

A *p*-value of 0.05 is considered significant; ** indicates *p* < 0.01, and * indicates *p* < 0.05.

**Table 4:** The instantaneous consumption rates (Day 4) and steady-state consumption rates of TN and TP at different flow velocities.

Flow Velocity (m/s)	Instantaneous Consumption Rate		Steady-state Consumption Rate	
	TN	TP	TN	TP
0	64.00	1.45	17.72	0.12
0.1	51.46	1.80	15.11	0.10
0.2	59.00	1.72	15.08	0.07
0.3	53.63	1.65	14.80	0.06
0.4	59.41	1.42	13.54	0.03
0.5	50.53	1.76	13.87	−0.12

**Table 5:** Correlations between AKP and other indexes at different flow velocities for 24 days.

Flow Velocity (m/s)	TP	Biomass	MDA	SOD
0	0.386	−0.617	0.44	0.412
0.1	0.323	−0.894 **	0.804 *	0.670
0.2	0.393	−0.966 **	0.976 **	0.772 *
0.3	0.460	−0.975 **	0.988 **	0.858 *
0.4	0.510	−0.970 **	0.922 **	0.896 **
0.5	0.394	−0.969 **	0.945 **	0.826 *

A *p*-value of 0.05 is considered significant; ** indicates *p* < 0.01, and * indicates *p* < 0.05.

## Supplementary Materials

The following are available online at https://www.mdpi.com/1660-4601/16/23/4641/s1, Figure S1: The correlation between biomass and Chla at different flow velocities, Figure S2: The correlation between MDA and biomass at different flow velocities, Figure S3: The correlation between MDA and SOD at different flow velocities, Table S1: Effects of different flow velocities on biomass, Table S2: Effects of different flow velocities on MDA, Table S3: Effects of different flow velocities on SOD, Table S4: Effects of different flow velocities on POD, Table S5: Effects of different flow velocities on CAT, Table S6: Effects of different flow velocities on TN, Table S7: Effects of different flow velocities on TP, Table S8: Effects of different flow velocities on AKP, Table S9: Effects of different flow velocities on pH.
