# Supplementary material for: Mechanism Underlying Flow Velocity and Its Corresponding Influence on the Growth of Euglena gracilis, a Dominant Bloom Species in Reservoirs
Source: Int J Environ Res Public Health. 2019 Nov 22;16(23):4641. doi: 10.3390/ijerph16234641 (PMC6926883; doi:10.3390/ijerph16234641)
Supplement: Supplementary file 1 [file ijerph-16-04641-s001.pdf]

Mechanism Underlying Flow Velocity and its Corresponding Influence on the Growth of *Euglena gracilis*, a Dominant Bloom Species in Reservoirs

Supplementary material

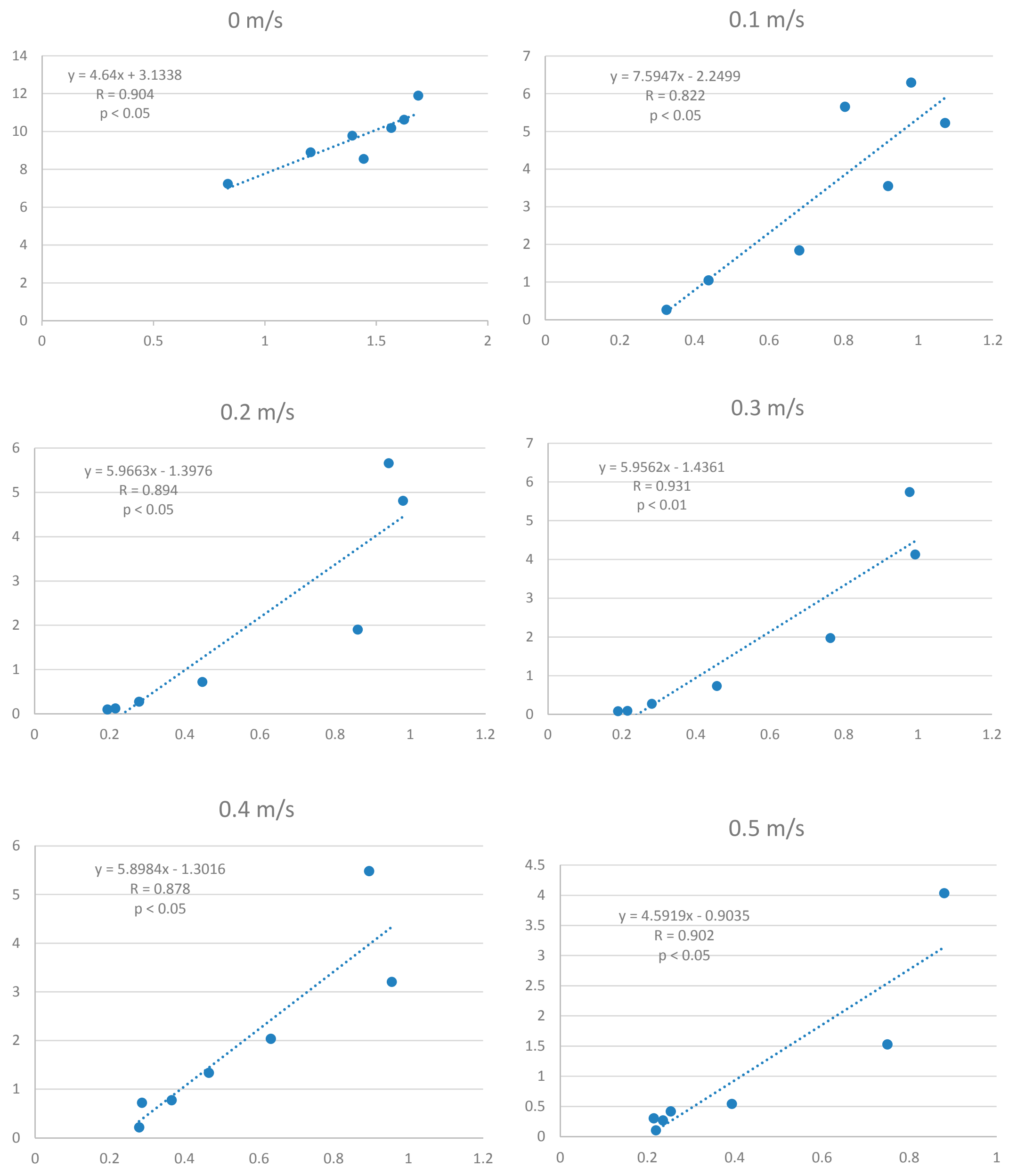

Figure S1. The correlation between biomass and Chla at different flow velocities.

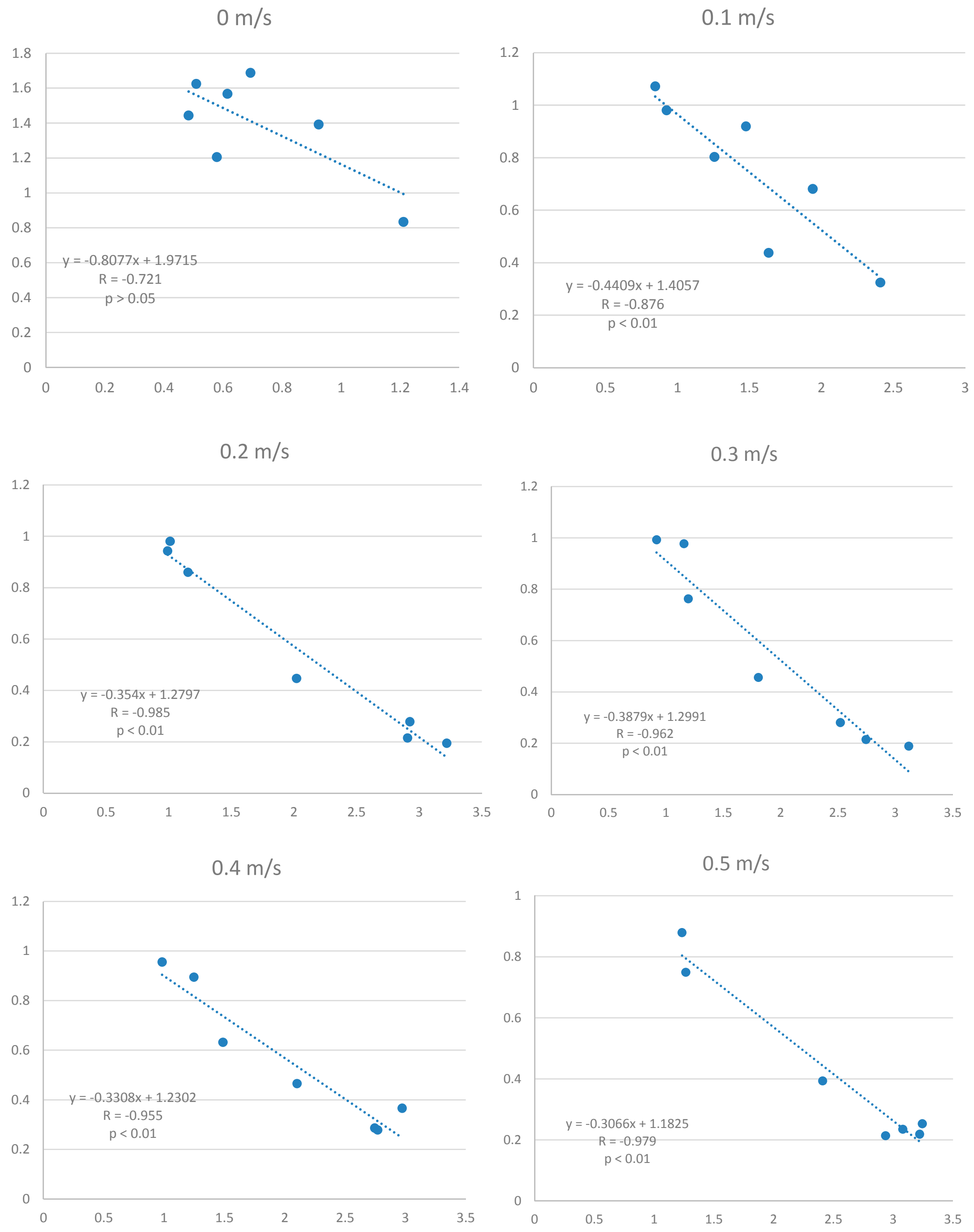

Figure S2. The correlation between MDA and biomass at different flow velocities.

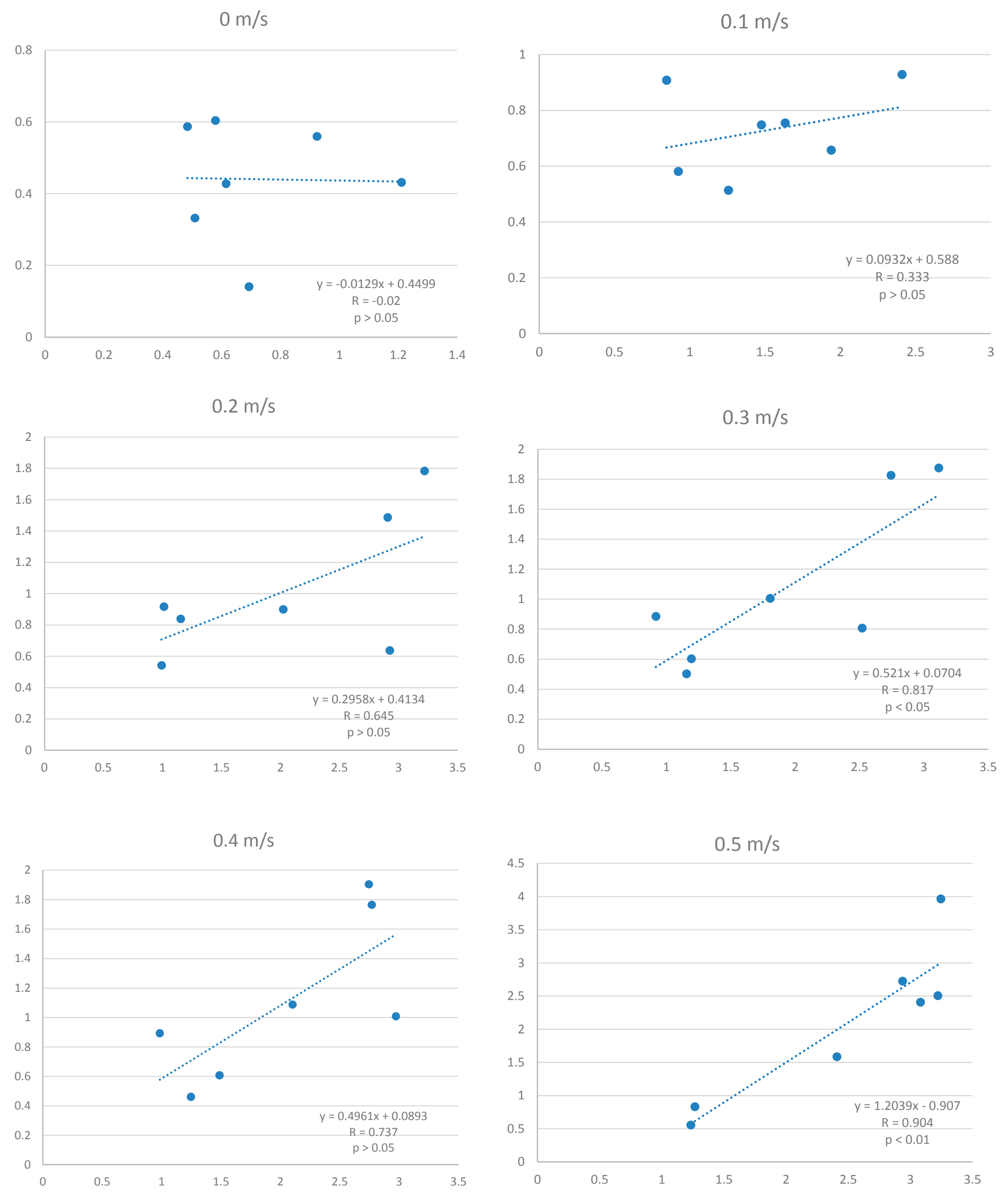

Figure S3. The correlation between MDA and SOD at different flow velocities. MDA: malondialdehyde; SOD: superoxide dismutase.

Table S1. Effects of different flow velocities on biomass.

Flow Velocity (m/s)	Biomass (10^8 cells/L)						
	**0**	**4**	**8**	**12**	**16**	**20**	**24**
0	0.83±0.023ab	1.2±0.072a	1.44±0.172a	1.39±0.141a	1.57±0.184a	1.62±0.031a	1.69±0.128a
0.1	0.8±0.066b	0.98±0.097b	1.07±0.093b	0.92±0.087b	0.68±0.077b	0.44±0.026b	0.32±0.028b
0.2	0.94±0.061ab	0.98±0.086b	0.86±0.077c	0.45±0.042c	0.28±0.026c	0.21±0.005d	0.19±0.017c
0.3	0.98±0.055a	0.99±0.075b	0.76±0.061cd	0.46±0.03c	0.28±0.03c	0.21±0.012c	0.19±0.002c
0.4	0.89±0.101ab	0.96±0.121b	0.63±0.022d	0.47±0.039c	0.37±0.043c	0.29±0.025d	0.28±0.02bc
0.5	0.88±0.082ab	0.75±0.055c	0.39±0.036e	0.25±0.021d	0.24±0.028c	0.21±0.015d	0.22±0.008bc

All values are the means of triplicates ± SD (n=3). ANOVA was significant at $p < 0.05$. Different letters indicate significantly different values for a particular treatment group (DMRT, $p < 0.05$).

Table S2. Effects of different flow velocities on MDA.

Flow Velocity (m/s)	MDA (nmol/10^8 cells)						
	**0**	**4**	**8**	**12**	**16**	**20**	**24**
0	1.21±0.047ab	0.58±0.029c	0.48±0.062e	0.92±0.019d	0.61±0.05d	0.51±0.027c	0.69±0.044c
0.1	1.26±0.1a	0.92±0.022b	0.85±0.043d	1.48±0.124c	1.94±0.17c	1.63±0.085b	2.41±0.176b
0.2	0.99±0.083b	1.01±0.061b	1.15±0.082c	2.02±0.267b	2.93±0.322ab	2.91±0.171a	3.22±0.301a
0.3	1.16±0.114ab	0.92±0.085b	1.19±0.065c	1.81±0.226bc	2.52±0.25b	2.74±0.336a	3.12±0.268a
0.4	1.25±0.126a	0.98±0.09b	1.49±0.066b	2.1±0.155b	2.97±0.15ab	2.75±0.273a	2.77±0.195ab
0.5	1.23±0.125ab	1.26±0.043a	2.41±0.162a	3.24±0.348a	3.08±0.302a	2.94±0.163a	3.22±0.202a

All values are the means of triplicates ± SD (n=3). ANOVA was significant at $p < 0.05$. Different letters indicate significantly different values for a particular treatment group (DMRT, $p < 0.05$).

Table S3. Effects of different flow velocities on SOD.

Flow Velocity (m/s)	SOD activity (units/10^8 cells)						
	**0**	**4**	**8**	**12**	**16**	**20**	**24**
0	0.43±0.035b	0.6±0.038b	0.59±0.067c	0.56±0.04d	0.43±0.037d	0.33±0.024e	0.14±0.004d
0.1	0.51±0.048ab	0.58±0.048b	0.91±0.099b	0.75±0.059cd	0.66±0.035cd	0.75±0.094d	0.93±0.015c
0.2	0.54±0.031ab	0.92±0.074a	0.84±0.045b	0.9±0.069bc	0.64±0.037cd	1.49±0.126c	1.78±0.185b
0.3	0.5±0.047ab	0.88±0.082a	0.6±0.026c	1.01±0.016bc	0.81±0.021bc	1.83±0.189bc	1.88±0.077b
0.4	0.46±0.062ab	0.89±0.12a	0.61±0.054c	1.09±0.082b	1.01±0.084b	1.9±0.122b	1.76±0.137b
0.5	0.56±0.057a	0.83±0.036a	1.59±0.077a	3.97±0.283a	2.41±0.255a	3.24±0.296a	2.34±0.215a

All values are the means of triplicates ± SD (n=3). ANOVA was significant at $p < 0.05$. Different letters indicate significantly different values for a particular treatment group (DMRT, $p < 0.05$).

Table S4. Effects of different flow velocities on POD.

Flow Velocity (m/s)	POD activity (units/10^8 cells)						
	0	4	8	12	16	20	24
0	377.79±10.849c	132.8±7.188d	44.15±1.528c	23.51±1.281e	36.92±3.84c	32.02±2.63d	80±5.628d
0.1	449.21±34.768ab	256.95±18.092c	21.48±1.375c	391.7±26.55b	40.65±1.163c	93.68±5.106c	138.56±7.538ab
0.2	435.16±27.574bc	296.54±7.916bc	72.46±4.96c	553.79±49.912a	75.53±4.135c	100.12±1.126c	111.12±13.206c
0.3	464.86±49.723ab	293.36±24.142bc	59.64±2.829c	497.17±41.897a	320.8±8.101b	144.12±10.233b	120.16±2.7bc
0.4	511.23±28.149a	318.21±8.935b	554.7±65.37b	193.39±17.253d	488.66±50.105a	157.33±9.954b	161.49±16.414a
0.5	454.69±10.376ab	480.2±42.654a	1257.49±41.161a	266.34±23.769c	497.17±33.314a	189.47±9.559a	148.6±12.592a

All values are the means of triplicates ± SD (n = 3). ANOVA was significant at $p < 0.05$. Different letters indicate significantly different values for a particular treatment group (DMRT, $p < 0.05$).

Table S5. Effects of different flow velocities on CAT.

Flow Velocity (m/s)	CAT activity (units/10^8 cells)						
	0	4	8	12	16	20	24
0	114.29±14.75b	139.44±14.109c	99.8±6.329e	22.04±1.222d	86.16±4.349d	81±5.742c	90±10.461c
0.1	280.1±11.797a	195.01±18.041c	223.91±13.264b	85.68±1.274cd	238.45±26.963c	184±4.475b	149.22±10.617b
0.2	111.36±15.027b	133.09±13.545c	136.07±3.904d	50.34±2.938cd	583.45±17.009b	55.82±3.599c	187.29±11.838a
0.3	92.09±7.432b	453.37±44.903b	141.55±8.453d	131.44±10.681c	174.98±4.577cd	77.57±7.849c	95.11±12.171c
0.4	76.94±3.216b	499.36±38.749b	199.45±4.676c	595.04±31.377b	191.39±16.15c	209.78±21.067ab	63.11±1.077d
0.5	288.22±32.339a	740.31±77.3a	469.3±16.893a	710.25±90.58a	1813.89±99.53a	218.89±19.3a	136.75±10.398b

All values are the means of triplicates ± SD (n=3). ANOVA was significant at $p < 0.05$. Different letters indicate significantly different values for a particular treatment group (DMRT, $p < 0.05$).

Table S6. Effects of different flow velocities on TN.

Flow Velocity (m/s)	TN (mg/L)						
	0	4	8	12	16	20	24
0	446.63±34.845a	190.65±13.506a	172.26±9.903b	153.86±16.636b	90.72±10.82b	56.06±2.698c	21.4±2.517d
0.1	416.63±36.936a	210.8±11.643a	215.35±23.102a	219.9±13.381a	68.71±6.025c	21.4±2.303d	53.95±2.851c
0.2	426.63±30.464a	190.65±8.274a	160.38±9.441b	130.11±11.433bc	200.21±15.673a	132.41±10.734b	64.62±7.613bc
0.3	406.63±32.466a	192.12±10.481a	126.73±4.49c	61.33±4.343e	51.33±5.583c	207.09±18.682a	51.33±4.961c
0.4	436.63±35.078a	198.99±11.74a	158.18±7.654b	117.37±11.937c	22.36±1.622e	66.97±4.216c	111.58±6.319a
0.5	406.61±29.511a	204.5±10.697a	146.16±14.116bc	87.82±2.292d	60.02±7.188c	46.48±3.526c	73.69±8.477b

All values are the means of triplicates ± SD (n=3). ANOVA was significant at $p < 0.05$. Different letters indicate significantly different values for a particular treatment group (DMRT, $p < 0.05$).

Table S7. Effects of different flow velocities on TP.

Flow Velocity (m/s)	TP (mg/L)						
	0	4	8	12	16	20	24
0	8.86±0.74a	3.06±0.21a	4.79±0.487a	3.49±0.281b	3.12±0.159c	5.11±0.445c	6.06±0.675b
0.1	9.11±0.667a	1.92±0.089bc	2.59±0.18d	3.09±0.206b	4.46±0.295b	6.19±0.703bc	6.65±0.381b
0.2	8.86±0.969a	1.97±0.178b	3.48±0.109c	4.66±0.394a	5.95±0.604a	6.62±0.687ab	7.29±0.45b
0.3	8.59±0.336a	1.99±0.146b	3.31±0.103c	5.13±0.068a	6.37±0.585a	7.16±0.611ab	7.23±0.49b
0.4	7.92±1.043a	2.26±0.152b	3.78±0.253bc	4.96±0.407a	6.15±0.579a	7.7±0.568a	7.16±0.629b
0.5	8.65±0.704a	1.6±0.096c	4.35±0.483ab	5.37±0.712a	6.44±0.893a	7.83±0.191a	11.6±1.199a

All values are the means of triplicates ± SD (n=3). ANOVA was significant at $p < 0.05$. Different letters indicate significantly different values for a particular treatment group (DMRT, $p < 0.05$).

Table S8. Effects of different flow velocities on AKP

Flow Velocity (m/s)	AKP synthase (units/10^8 cells)						
	0	4	8	12	16	20	24
0	0.05±0.005a	0.05±0.004a	0.05±0.001e	0.04±0.003e	0.04±0.002d	0.04±0.005d	0.04±0.001d
0.1	0.05±0.006a	0.05±0.004a	0.09±0.009d	0.09±0.006d	0.11±0.004c	0.19±0.01c	0.25±0.029c
0.2	0.05±0.003a	0.05±0.001a	0.11±0.008cd	0.21±0.026c	0.31±0.009b	0.42±0.032b	0.45±0.036b
0.3	0.05±0.004a	0.05±0.006a	0.12±0.005c	0.22±0.019c	0.31±0.02b	0.41±0.033b	0.44±0.015b
0.4	0.05±0.003a	0.05±0.005a	0.19±0.012b	0.28±0.024b	0.34±0.026b	0.49±0.028b	0.49±0.058b
0.5	0.05±0.002a	0.05±0.003a	0.4±0.02a	0.55±0.039a	0.62±0.059a	0.74±0.071a	0.58±0.018a

All values are the means of triplicates ± SD (n=3). ANOVA was significant at $p < 0.05$. Different letters indicate significantly different values for a particular treatment group (DMRT, $p < 0.05$).

Table S9. Effects of different flow velocities on pH.

Flow Velocity (m/s)	pH						
	0	4	8	12	16	20	24
0	7.44±0.3	8.22±0.27	8.22±0.26	8.26±0.28	8.3±0.31	8.33±0.4	8.37±0.23
0.1	7.3±0.46	8.27±0.32	8.47±0.29	8.54±0.23	8.62±0.19	8.69±0.17	8.76±0.36
0.2	7.43±0.3	8.28±0.14	8.55±0.21	8.61±0.38	8.66±0.37	8.72±0.46	8.77±0.44
0.3	7.47±0.47	8.28±0.38	8.56±0.51	8.64±0.53	8.72±0.3	8.79±0.33	8.87±0.43
0.4	7.43±0.47	8.51±0.34	8.71±0.12	8.75±0.36	8.8±0.12	8.84±0.21	8.88±0.3
0.5	7.81±0.38	8.65±0.23	8.72±0.23	8.77±0.28	8.82±0.5	8.86±0.09	8.91±0.33

All values are the means of triplicates ± SD (n=3).
